# Cyr61 synthesis is induced by interleukin-6 and promotes migration and invasion of fibroblast-like synoviocytes in rheumatoid arthritis

**DOI:** 10.1186/s13075-020-02369-8

**Published:** 2020-11-23

**Authors:** Changmin Choi, Wooseong Jeong, Byeongzu Ghang, Yonggeun Park, Changlim Hyun, Moonjae Cho, Jinseok Kim

**Affiliations:** 1 Department of Medicine, Jeju National University School of Medicine, Jeju, Republic of Korea; 2 Department of Internal Medicine, Division of Rheumatology, Jeju National University Hospital, Aran 13gil, Jeju, 690-797 Republic of Korea; 3 Department of Orthopaedic Surgery, Jeju National University Hospital, Jeju, Republic of Korea; 4 Department of Pathology, Jeju National University Hospital, Jeju, Republic of Korea; 5 Department of Biochemistry, Jeju National University School of Medicine, Aran 13gil, Jeju, 690-797 Republic of Korea

**Keywords:** Cyr61, Interleukin-6, Extracellular signal-regulated kinase, Fibroblast-like synoviocyte, Rheumatoid arthritis

## Abstract

**Background:**

Interleukin-6 (IL-6) is involved in fibroblast-like synoviocyte (FLS) activation and promotes pannus formation and bone and cartilage destruction in rheumatoid arthritis (RA). Cysteine-rich 61 (Cyr61) protein regulates cell proliferation, migration, and differentiation. The aim of this study was to investigate the role of Cyr61 in RA-FLS migration and invasion after IL-6 stimulation.

**Methods:**

Western blotting, immunohistochemistry, reverse transcription-polymerase chain reaction, and real time-polymerase chain reaction were used to examine protein and mRNA levels of Cyr61, matrix metalloproteinases (MMPs), and other signalling proteins. Knockdown of gene expression was performed with siRNA, and RNA sequencing was performed for differential gene analysis. Migration and invasion were assessed by wound healing and Boyden chamber assays.

**Results:**

Cyr61 levels were elevated in FLSs from RA patients compared to those in osteoarthritis patients. Control and IL-6-treated FLSs showed differential gene expression. IL-6 stimulated protein synthesis of Cyr61, which was attenuated by the extracellular signal-related kinase 1/2 (ERK 1/2) inhibitor, PD98059, and knockdown of early growth response 3 (*EGR3*), but not of *JUN*. IL-6-induced Cyr61 protein synthesis increased expression of *MMP2*. Cyr61 promoted FLS migration and invasion in an autocrine manner. Knockdown of *CYR61* and a neutralising antibody attenuated Cyr61 synthesis and IL-6-induced FLS migration.

**Conclusions:**

By modulating the ERK/EGR3 pathway, IL-6 stimulated Cyr61 production and in turn increased invasiveness of FLS. Our data suggest that Cyr61 might be a potential target to prevent the progression of joint damage in RA.

## Background

Rheumatoid arthritis (RA) is a chronic inflammatory disease that causes destruction of cartilage and bone and systemic inflammation via the interactions of different types of inflammatory cells [1, 2]. Fibroblast-like synoviocytes (FLSs) play an important role in the pathogenesis of RA and are major components of the hyperplastic pannus that invades cartilage and bone. These cells contribute to the local production of pro-inflammatory cytokines and enzymes that degrade the extracellular matrix (ECM) [3]. In addition, RA FLSs present a tumour-like phenotype with increased invasiveness into the extracellular matrix, which further exacerbates synovial hyperplasia and joint damage. These processes that are important for migration and invasion are mediated by cytoskeletal movement and the expression of adhesion proteins and proteolytic enzymes [4].

Cysteine-rich protein 61 (Cyr61 or CCN1) is an ECM component that belongs to the CCN family consisting of six members, CCN1–CCN6 [5]. Cyr61 mediates cell proliferation, adhesion, migration, and differentiation. The role of Cyr61 has been extensively studied in tumour biology and is also considered important for RA [6, 7]. Cyr61 is stimulated by interleukin-17 (IL-17) and, in turn, promotes FLS proliferation, thus contributing to the hyperplasia of synoviocytes [8].

IL-6 is a pro-inflammatory cytokine that triggers host defence by sending out inflammatory signals when microbial infections or tissue damage occur. These responses are critical for the elimination of pathogens and regeneration of injured tissues. However, persistence of IL-6 stimulates the onset of inflammatory and auto-immune diseases such as diabetes, systemic lupus erythematosus, and RA [9]. IL-6 signals through binding to the membrane-bound IL-6 receptor (mIL-6R) via the classic signalling pathway or to the soluble IL-6 receptor (sIL-6R) via the trans-signalling pathway. After IL-6 binds to mIL-6R or sIL-6R, a cell surface glycoprotein called gp130 is recruited to form a receptor complex with IL-6 and IL-6R. This complex triggers downstream signalling and generates various biological responses [10]. IL-6 levels are elevated in the synovial fluid (SF) and sera of RA patients, suggesting that IL-6 mediates many of the local and systemic effects of this disease. IL-6 is involved in FLS activation, osteoclast activation affecting pannus formation, and bone and cartilage destruction [11].

In this study, we investigated whether and how IL-6 stimulates the protein synthesis of Cyr61 and contributes to the invasion and migration of RA FLS. Further, we explored how Cyr61 affects FLSs in the development of RA.

## Methods

### Isolation and culture of primary FLSs

Primary FLSs of RA patients (*n* = 5) were obtained from Samsung Medical Center and primary synovial tissues of RA patients (*n* = 4) and osteoarthritis (OA) patients (*n* = 4) from Jeju National University Hospital for comparison. The study was approved by the Institutional Review Board of Jeju National University. Informed consent in accordance with the Declaration of Helsinki was obtained from all patients.

To isolate primary FLSs, primary synovial tissues were cut into pieces with operating scissors, digested in collagenase (Life Technologies, Grand Island, NY, USA) and dispase (Life Technologies, Grand Island, NY, USA) and dissolved in Eagle’s Minimum Essential Medium (EMEM) (BioWhittaker, Inc., Walkersville, MD, USA). The medium containing the minced tissues was filtered through nylon mesh and centrifuged at 1500 rpm, 5 min, and 23 °C. The cells were cultured in Dulbecco’s MEM (DMEM) (BioWhittaker, Inc., Walkersville, MD, USA) supplemented with 10% foetal bovine serum (FBS) (Merck KGaA, Darmstadt, Germany) and primocin (InvivoGen, San Diego, CA, USA) in a humidified 5% CO_2_ atmosphere. Cells from the 4th to 8th generations were used for experiments.

### Reagents

IL-6, soluble IL-6 receptor, and CYR61 protein were purchased from PeproTech, Inc. (Rocky Hill, NJ, USA). Monoclonal antibodies against human extracellular signal-regulated kinases 1/2 (ERK 1/2), phospho-ERK 1/2, Cyr61, beta-actin, and early growth response protein 3 (EGR3), Nuclear receptor subfamily 4 group A member 1 (NR4A1), Activating Transcription factor 3 (ATF3) as well as anti-mouse and anti-rabbit secondary antibodies were from Santa Cruz Biotechnology, Inc. (Santa Cruz, CA, USA). Antibodies against c-Jun were purchased from Cell Signaling (Danvers, MA, USA). Cyr61-neutralising antibody was purchased from Novus Biologicals (Littleton, CO, USA).

### Western blotting and culture supernatants

Cells were washed twice with phosphate-buffered saline (PBS) and lysed with radioimmunoprecipitation assay buffer (Thermo Fisher Scientific, Rockford, IL, USA) containing Proteinase Inhibitor (BioVision, Milpitas, CA, USA). Cell lysates were collected by scraping and centrifuged at 14,000 rpm for 15 min at 4 °C. Protein concentrations were determined by using a bicinchoninic acid assay kit (Thermo Fisher Scientific, Rockford, IL, USA). Equal amounts (25 μg) of proteins from all samples were electrophoresed on 10% sodium dodecyl sulphate-polyacrylamide gels and transferred to nitrocellulose membranes that were subsequently blocked for 1 h with 5% non-fat milk in tris (hydroxymethyl) aminomethane (Tris)-buffered saline (TBS) containing 0.1% Tween 20 (TBST). The membranes were incubated overnight at 4 °C with specific primary antibodies. After washing with PBS, the membranes were incubated with horseradish peroxidase-conjugated secondary antibodies at room temperature for 1 h, followed by washing with PBS. The target proteins were examined with an enhanced chemiluminescence reagent (CYANAGEN, Bologna, Italy) and detected with autoradiography film.

To examine the effects of IL-6 on Cyr61 production in FLSs, FLSs were cultured with IL-6 for 24 h, and the culture supernatants were collected. Culture medium was concentrated onto Amicon Ultra 2 membranes (Merck KGaA, Darmstadt, Germany) at 4 °C according to the manufacturer’s protocol. Cyr61 protein levels in the cell culture supernatants were determined by western blotting.

### RNA extraction and reverse transcription-polymerase chain reaction (RT-PCR)

Total RNA was extracted from FLSs using TRIzol (Invitrogen, Carlsbad, CA, USA) according to the manufacturer’s protocol. cDNA was synthesised by using a reverse transcriptase kit (Promega, Seoul, Korea). PCR primers for human matrix metalloproteinase 1 (*MMP1*), *MMP2*, *CYR61*, and glyceraldehyde 3′-phosphate dehydrogenase (*GAPDH*) were as follows: *MMP1* forward (5′-GGAGATCATCGGGACAACTC-3′), *MMP1* reverse (5′-ACCGGACTTCATATGTCG-3′), *MMP2* forward (5′-GAACACAGCCTTCTCCTCCT-3′), *MMP2* reverse (5′-CATCAAGGGCATTCAGGAGC-3′), *CYR61* forward (5′-TCCTCTGTGTCCCCAAGAAC-3′), *CYR61* reverse (5′-TCGAATCCCAGCTCCTTTACC-3′), *GAPDH* forward (5′-CCAAGGAGTAAGACCCCTGG-3′), *GAPDH* reverse (5′-TGGTTTGAGCACAGGGTACTT-3′). PCR products were loaded on a 1% agarose gel. Differences in band intensity were confirmed using ImageJ software (NIH, MD, USA) to analyse the relative levels in target RNAs.

### Real-time polymerase chain reaction

Total RNA extraction and cDNA synthesis were performed as previously described. Real-time PCR was performed using SYBR Green Master mix (KAPA BIOSYSTEMS, Cape Town, South Africa) according to the manufacturer’s instructions. The primers for human *MMP1*, *MMP2*, *CYR61*, *GAPDH* were as follows; *MMP1* forward (5′-GGTAGAGCGTTCTAGGTGTATG-3′), *MMP1* reverse (5′-AACCCTCTGGCTAGAAGTAGTC-3′), *MMP2* forward (5′-AACCCTCTGGCTAGAAGTAGTC-3′), *MMP2* reverse (5′-CCTGTAGAGTTCACTCCTTACG-3′), *CYR61* forward (5′-GACCTGTGGAACTGGTATCTC-3′), *CYR61* reverse (5′-CCAGCGTAAGTAAACCTGAC-3′), *IL-6* forward (5′-CCTAGAGTACCTCCAGAACAGA-3′), *IL-6* reverse (5′-CATTTGTGGTTGGGTCAG-3′), *GAPDH* forward (5′-CACAAGAGGAAGAGAGAGACC-3′), *GAPDH* reverse (5′-CCTCTTCAAGGGGTCTACAT-3′).

### RNA interference (RNAi) for knockdown of gene expression

*CYR61*, *EGR3*, *c*-*Jun*, NR4A1, and ATF3 small interfering RNAs (siRNAs) were designed and synthesised at Bioneer Corp (Dajeon, Korea). Briefly, cells were seeded in 60-mm dishes at a density of 3 × 10^5^ cells/dish and incubated overnight. After aspiration of the medium, a transfection mixture of siRNA oligonucleotides and Lipofectamine 2000 reagent (Invitrogen, Carlsbad, CA, USA) in serum-free medium was added to the cells and incubated for 4 h. The medium was replaced with DMEM containing 10% FBS and incubated for an additional 24-h period.

### Cell invasion and migration analysis

FLSs were seeded at a density of 10^5^ cells/well in six-well plates. After 24 h of incubation when the cells were 70–80% confluent, the cell monolayers were scratched with a 200-μL pipet tip. Scratched monolayers were washed with PBS to remove detached cells. The bottom of the dish was marked for reference. The wound area was recorded during the 24-h period, and the images acquired for each sample were analysed quantitatively using ImageJ software. Wound closure rate was calculated as {(initial area − final area)/initial area} × 100.

For the invasion assay, transwells (Corning Incorporated, Corning, NY, USA) were used into which cells in serum-free medium were added to the upper chambers. The lower chambers of the transwells contained medium supplemented with 20% FBS and Cyr61 protein with or without anti-Cyr61 antibody. After 24 h of incubation, non-invaded cells remained above membranes of upper chamber were carefully removed with a cotton swab. Cells that had invaded into the underside of the membrane were fixed with 4% paraformaldehyde and stained with crystal violet solution. The number of cells stained was calculated by visually counting three randomly chosen areas. All experiments were performed in triplicate.

### Transcriptome analysis by RNA sequencing and analysis of RNA-seq data

Total RNA isolation was performed as described in the RT-PCR section. cDNA synthesis, sequencing, and analysis of RNA-seq data were conducted by Cosmo Gentech. Co (Seoul, Korea). Differentially expressed genes (DEGs) were analysed by using online tools in Metascape (http://metascape.org). Functional enrichment was performed in cellular component (CC), molecular function (MF), and biological process (BP). Kyoto Encyclopedia of Genes and Genomes (KEGG) pathway enrichment was also performed.

### Cell proliferation analysis

Cell proliferation analysis was performed via Electric Cell-substrate Impedance Sensing (ECIS). Electrode-stabilising solution (200 uL) containing 10 mM L-cysteine (Applied Biosystems, Jordan Road Troy, NY, USA) was added to each well of ECIS Cultureware 8W10E+ Polyethylene terephthalate (PET) (Applied Biosystems), which was then kept at room temperature for 10 min. Later, the wells were washed with distilled water (DW) and the cells (3 × 10^3^ cell/well) were seeded in each well. After 24 h, the cells were treated with IL-6/sIL-6R and Cyr61 protein and incubated at 37 °C.

### Immunohistochemistry (IHC)

Immunohistochemistry was performed on 4-μm-thick sections obtained from TMA blocks. The tissues sections were stained with mouse monoclonal anti-IL-6 antibody at a dilution of 1:400 (Santa Cruz, CA, USA) and rabbit polyclonal anti-Cyr61 antibody at a dilution of 1:200 (Santa Cruz, CA, USA) using an automated immunostainer (Benchmark Ultra, Ventana Medical Systems Inc., Tucson, AZ, USA). Glandular cells in colon tissue served as positive control for Cyr61 and a subset of cells in lymph node served as positive control for IL-6. The primary antibody was omitted for negative control.

### Statistical analysis

All experiments were repeated at least three times. Significance of differences was tested using analysis of variance, followed by Dunnett’s test or Tukey’s test (GraphPad Prism 8.0). *p* values < 0.05 were considered to be significant.

## Results

### Increase in Cyr61 protein synthesis in the FLSs of RA patients induced by IL-6

**Fig. 1:**
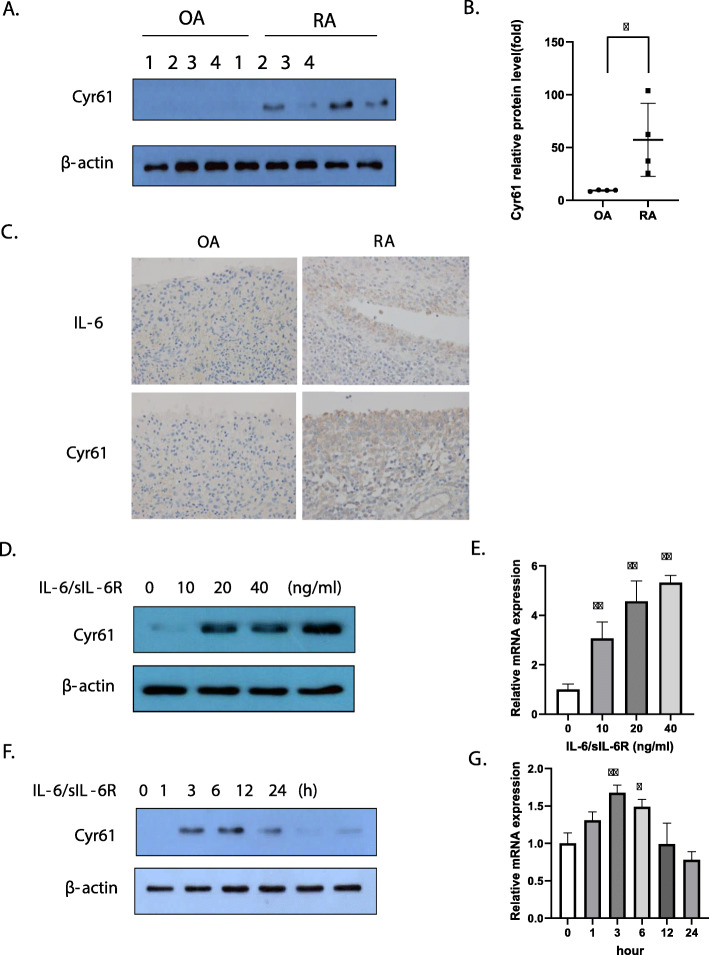
Expression of IL-6 and Cyr61 in fibroblast-like synoviocytes (FLSs). **a**, **b** FLSs from osteoarthritis (OA; *n* = 4) or rheumatoid arthritis (RA, *n* = 4) patients. *p < 0.05 vs OA. **c** Synovial tissues from OA or RA patients. Original magnification × 400. **d**, **e** RA FLSs stimulated by IL-6/sIL-6R for 2 h. **f**, **g** FLSs stimulated by IL-6/s IL-6R (20 ng/mL) for the indicated periods. **d**–**g** FLSs were incubated overnight in 1% FBS-containing medium before treatment with IL-6/sIL-6R. **a**, **b**, **d**, **f** Protein levels were determined by western blotting. **e**, **g** The mRNA levels of *Cyr61* were determined through real time polymerase chain reaction. Values are means (± standard deviation) of at least three independent experiments. *p < 0.05, **p < 0.01 vs untreated cells. IL-6, interleukin-6; sIL-6R, soluble IL-6 receptor; FBS, foetal bovine serum

As FLSs are involved in the pathogenesis of RA, and Cyr61 contributes to cell adhesion and migration, we first examined protein levels of Cyr61 in OA patients (*n* = 4) and RA patients (*n* = 4). We observed that Cyr61 protein levels were higher in FLSs from RA patients than in those from OA patients (Fig. 1a, b). As IL-6 is an important pro-inflammatory cytokine produced by lymphoid and non-lymphoid cells, such as T cells, B cells, monocytes, endothelial cells, and is found at high levels in the joint fluid [9, 11], we analysed IL-6 and Cyr61 protein level in synovial tissues. The results showed that IL-6 and Cyr61 protein levels were higher in RA synovial tissues than in OA synovial tissues. (Fig. 1c) Next, we examined the effects of IL-6 on Cyr61 protein synthesis. Because FLSs do not express mIL-6Rs, we treated the FLSs with equal concentrations of IL-6 and sIL-6Rs. The results revealed that Cyr61 mRNA expression and protein synthesis were enhanced in a dose- and time-dependent manner after IL-6 stimulation (Fig. 1d–g). Cyr61 mRNA expression and protein levels peaked at 20 ng/mL of IL-6 at 2 h post-IL-6 addition, followed by a gradual decline.

### Regulation by IL-6 of expression of genes associated with migration and mitogen-activated protein kinase (MAPK) responses

**Fig. 2:**
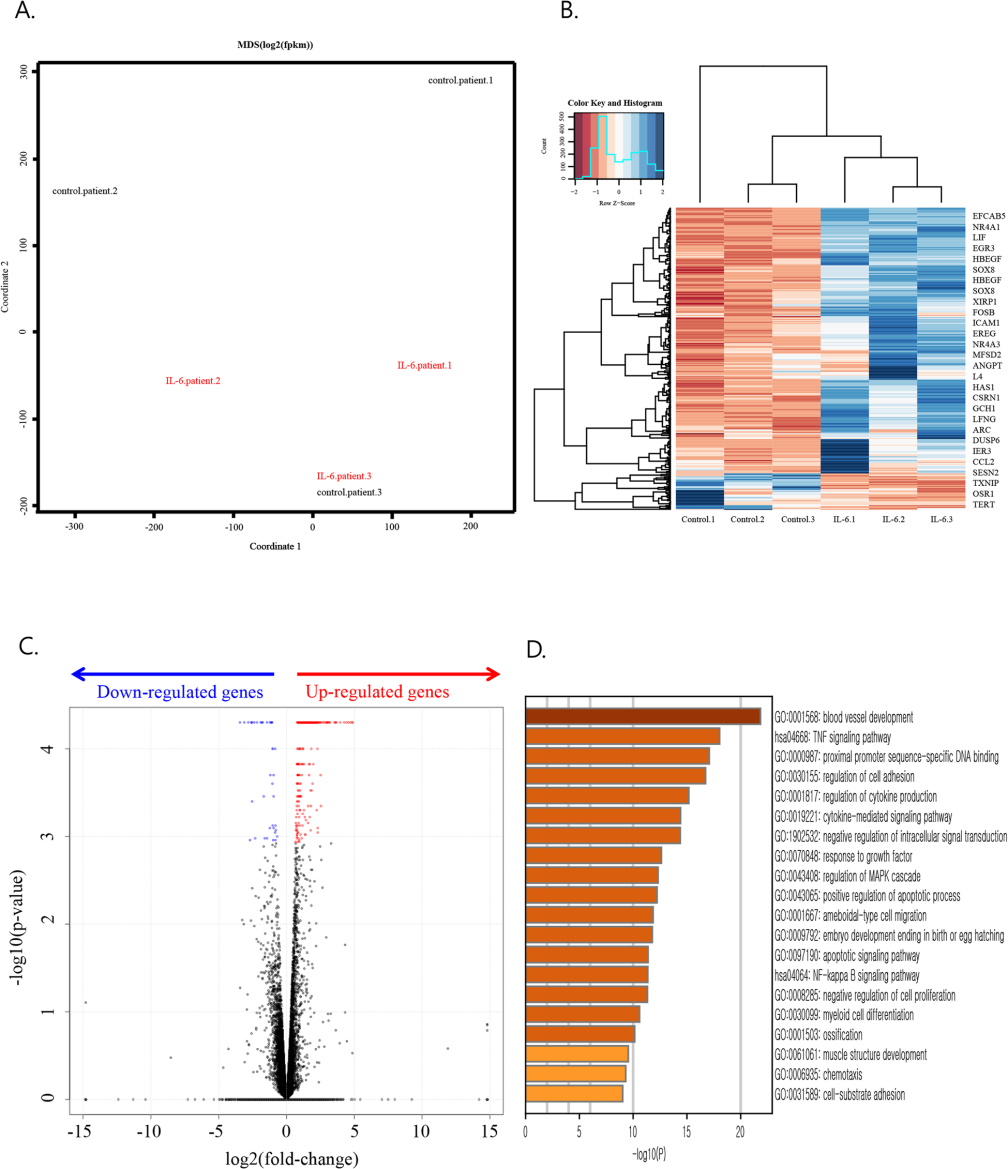
RNA sequencing of fibroblast-like synoviocytes from 3 rheumatoid arthritis patients and interleukin-6 (IL-6)-treated samples. Group 1 (control_patient 1, control_patient 2, control_patient 3), group 2 (IL-6_patient 1, IL-6_patient 2, IL-6_patient 3). **a** Multi-dimensional scaling (MDS) plot showing Pearson’s correlation coefficient between log2 (fragments per kilobase of exon per million fragments mapped; FPKM) of genes. **b** Heat map of differentially expressed genes. Gene expression expressed as FPKM. Expression normalised by z-score transformation before visualisation with heatmap. **c** Volcano plot of differentially expressed genes. The most upregulated genes are towards the right and the statistically significant genes are in red, the most downregulated genes are towards the left and the statistically significant genes are in blue, and the most statistically significant genes are towards the top. **b**, **c** Cutoff *q*-value < 0.05. **d** Top 20 Gene Ontology (GO) enrichment results after IL-6 treatment. Upregulated genes in 2B were subjected to GO analysis. Top 20 GO categories are shown (−log10 (*P*) values were calculated by Metascape software); has, *Homo sapiens*

To investigate the genome-wide effects of IL-6 on RA FLSs, transcriptome analysis by RNA-sequencing was performed using FLSs from 3 RA patients as a control group (group 1) and IL-6-treated FLSs from each patient (group 2). A multi-dimensional scaling (MDS) plot demonstrates the clustering of the transcriptomes of the three IL-6-treated samples, whereas no clustering was noted for the three untreated samples. These results indicate that gene expression increased in RA FLSs after IL-6 treatment (Fig. 2a). We identified a total of 315 DEGs, including 277 upregulated genes and 38 downregulated genes, by comparing group 1 and group 2 transcriptomes (Fig. 2b, c). Gene Ontology (GO) enrichment analysis using Metascape software revealed that these IL-6 induced DEGs were mainly involved in blood vessel development, tumour necrosis factor (TNF) signalling, and proximal promoter sequence-specific DNA binding. We also noticed that GOs involved in the regulation of the MAPK cascade and cell migration, including the regulation of cell adhesion, ameboidal-type cell migration, chemotaxis, and cell-substrate adhesion, are significantly increased by IL-6. (Fig. 2d) Furthermore, the DEGs downregulated by IL-6 were mainly involved in Forkhead box O (FOXO)-mediated transcription, regulation of the G1/S transition of the mitotic cell cycle, cellular response to glucose starvation, and cell fate commitment (data not shown).

### IL-6-induced Cyr61 production depends on ERK 1/2–EGR3 pathway

**Fig. 3:**
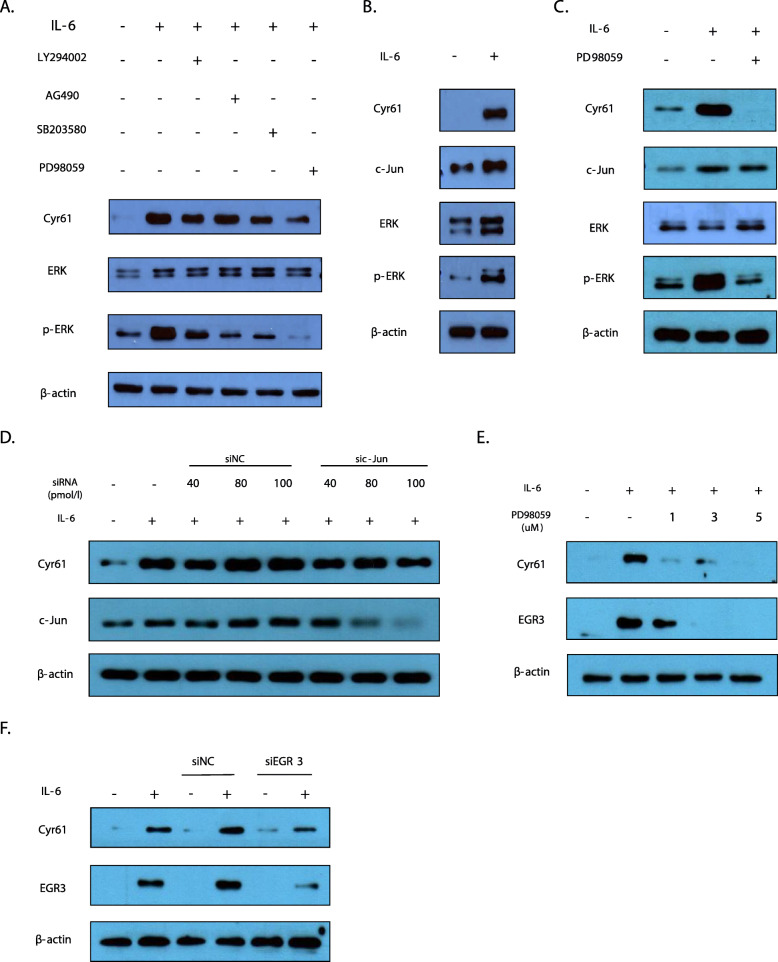
Signalling pathways involved in IL-6-regulated protein synthesis of Cyr61 in rheumatoid arthritis-fibroblast-like synoviocytes (RA-FLSs). **a**, **c**, **e** Cells were pretreated with inhibitors for 2 h before IL-6 (20 ng/mL) stimulation for 2 h. LY294002 (10 μM): PI3K/AKT inhibitor, AG490 (50 μM): JAK2/STAT3 inhibitor, SB203580 (10 μM): p38 MAPK inhibitor, PD98059 (1 μM): ERK inhibitor. **d**, **f** RA-FLSs transfected with either small interfering RNA (c-Jun or EGR3) or siNC (control) (20 pmol/L) stimulated by IL-6 (20 ng/mL) for 2 h. Data are representative of at least three independent experiments. **a–f** Protein levels were determined by western blotting. FLSs were incubated overnight in 1% FBS-containing medium before treatment with IL-6/sIL-6R

To explore which signalling pathways are responsible for IL-6-induced synthesis of Cyr61 protein, we used known inhibitors of several pathways, including LY294002 (an inhibitor of phosphoinositide 3-kinase (PI-3 K) activation), SB203580 (an inhibitor of p38 MAPK), PD98059 (an inhibitor of ERK1/2), and AG490 (an inhibitor of Janus-activated kinase 2 (JAK2)/signal transducer and activator of transcription 3 (STAT3)). IL-6-stimulated synthesis of Cyr61 protein was markedly decreased in the presence of the ERK 1/2 inhibitor. In contrast, other inhibitors showed little effect on IL-6-induced Cyr61 protein levels (Fig. 3a) Thus, these results indicate a dominant role for the non-canonical ERK 1/2 pathway in the regulation of Cyr61 protein synthesis by IL-6.

After analysis of RNA-sequencing, we found several transcriptional factors that might be involved in Cyr61 transcriptional regulation such as Nuclear receptor subfamily 4 group A member 1 (NR4A1), Activating Transcription factor 3 (ATF3), EGR3, and c-Jun. We examined these factors to determine their effects on Cyr61 protein synthesis.

In response to cytokines, growth factors, and oxidative stress, c-Jun binds to the promoters of target genes to modulate their expression in cell proliferation and inflammation in different types of cells [12, 13]. In this study, we confirmed that IL-6 stimulation increased c-Jun protein synthesis (Fig. 3b). We also found that the ERK 1/2 inhibitor PD98059 abrogated IL-6-induced Cyr61 protein synthesis (Fig. 3c, e). However, c-Jun protein synthesis was not affected by the ERK 1/2 inhibitor (Fig. 3c), and the knockdown of *c-Jun* expression using siRNA did not affect Cyr61 protein synthesis (Fig. 3d). Moreover, other transcription factors such as *NR4A1* and *ATF3* did not affect Cyr61 protein synthesis (Supplementary Fig. 1).

Interestingly, we found that EGR3 protein synthesis was regulated by ERK 1/2 (Fig. 3e). To examine the effect of EGR3 on Cyr61 protein synthesis, we knocked down *EGR3* expression using siRNA and observed a decrease in Cyr61 protein levels (Fig. 3f). These results indicate that EGR3 modulated Cyr61 protein synthesis through ERK 1/2 after IL-6 stimulation.

### IL-6 induced increase in autocrine Cyr61 protein production from FLSs

**Fig. 4:**
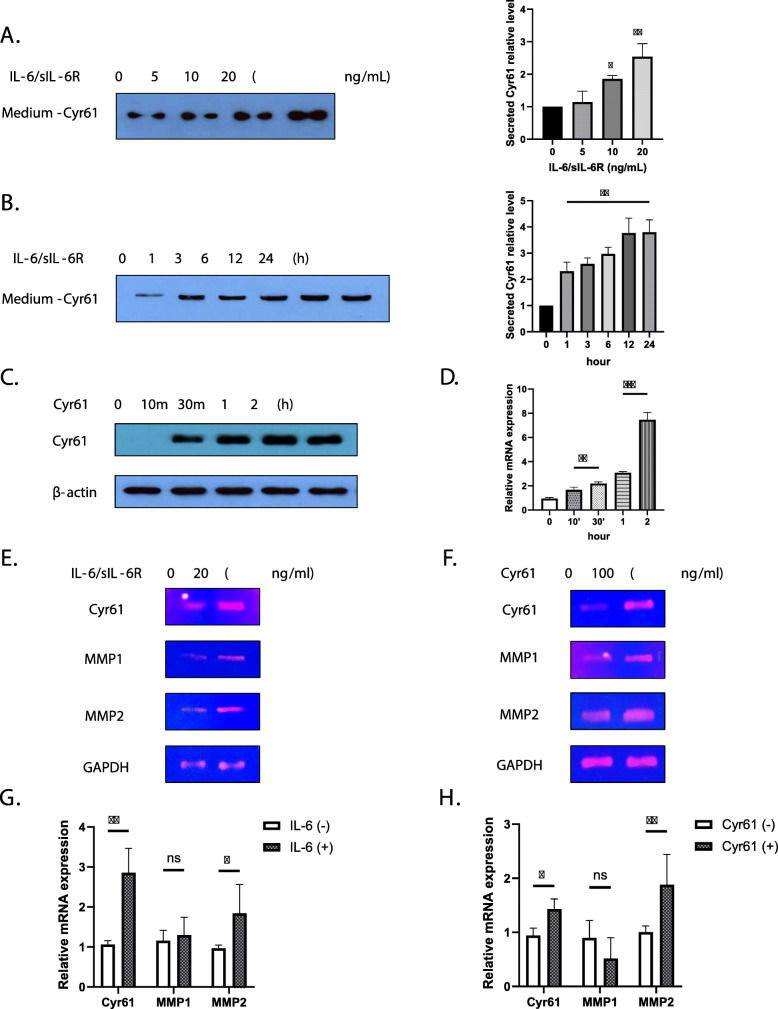
Cyr61 secretion induced by IL-6. **a**, **b** Extracellular protein levels of Cyr61 in culture supernatants of IL-6-treated RA-FLSs measured by western blotting. **b** IL-6 (20 ng/mL). **c**, **d** FLSs stimulated by extracellular Cyr61 (100 ng/mL) for indicated time periods. **c** Protein levels were determined by western blotting. **d** The mRNA levels of *Cyr61* were determined through real time polymerase chain reaction. **e**–**h** The mRNA levels of *Cyr61*, *MMP1*, *2*, and *GAPDH* induced by IL-6 (20 ng/mL) and extracellular Cyr61 protein (100 ng/mL) for 2 h. **e**, **f** The mRNA levels were determined by reverse-transcription polymerase chain reaction. **g**, **h** The mRNA levels were determined through real time polymerase chain reaction. Data are representative of at least three independent experiments. *p < 0.05, **p < 0.01, ***p < 0.001

Given that Cyr61 is an ECM component, we collected culture supernatants and measured the concentrations of secreted Cyr61. Cyr61 protein levels were increased by IL-6 in a time- and dose-dependent manner as shown in Fig. 1 (Fig. 4a, b). We then examined whether Cyr61 protein had an autocrine effect on the FLSs. The results show that Cyr61 protein synthesis and mRNA expression were increased by Cyr61 in the supernatant medium (Fig. 4c, d). Moreover, Cyr61 protein increased IL-6 mRNA level (Supplementary Fig. 2). Because MMPs are associated with joint destruction, cell migration, and invasion [14], we assessed the effects of IL-6 and Cyr61 protein on MMP protein synthesis. As demonstrated in Fig. 4e, f, g, and h, both IL-6 and Cyr61 increased *MMP2* mRNA levels. However, neither IL-6 nor Cyr61 protein affected *MMP1* expression. These results indicate that the induction of the expression of the *MMP* genes was partly dependent on IL-6 and Cyr61 protein.

### IL-6 stimulated RA-FLS migration and invasion through Cyr61 protein secretion

**Fig. 5:**
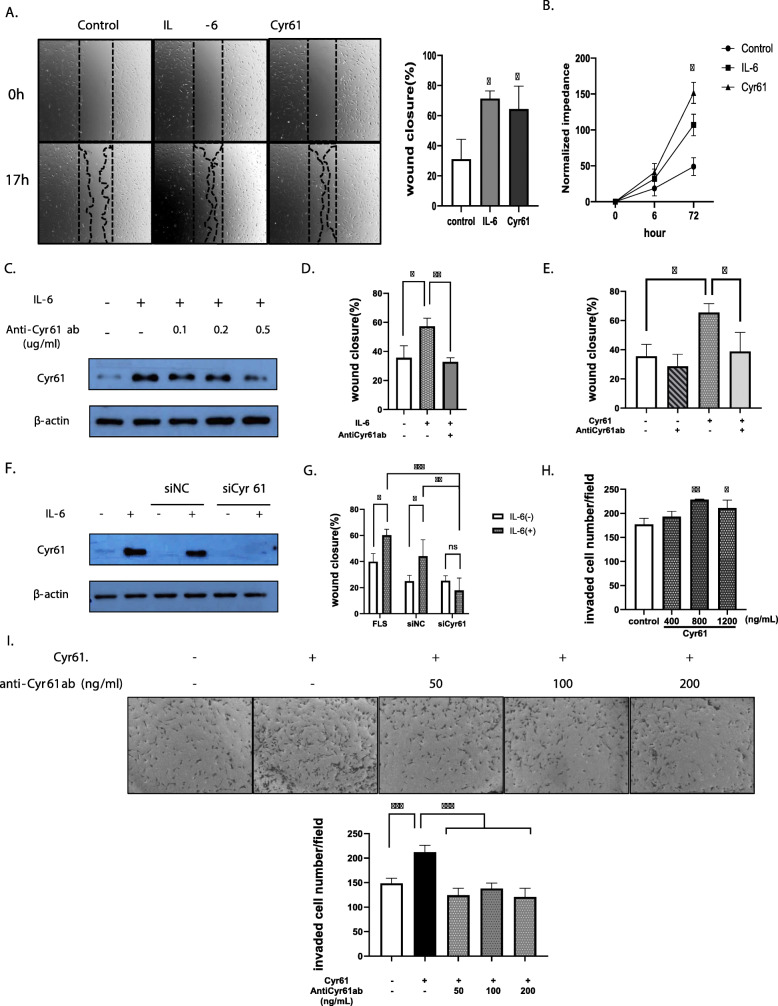
Migration and invasion of rheumatoid arthritis-fibroblast-like synoviocytes (RA-FLSs) promoted by IL-6 and Cyr61 secretion. **a**, **d**, **e**, **g** Wound-closure over 17 h. **b** ECIS proliferation analysis over 72 h. **c**, **f** Western blotting for Cyr61 protein detection. **h**, **i** Cyr61-stimulated invasion of RA-FLSs in transwells over 24 h ± antiCyr61 ab. **f**, **g** transfection with 20 pmol/L of small-interfering Cyr61 RNA (siCyr61) or siNC (negative control). IL-6 for 17 h (**a**, **d**, **g**: 200 ng/mL); IL-6 for 2 h (**c**, **f**: 20 ng/mL); IL-6 and Cyr61 protein for 72 h (**b**: IL-6: 200 ng/mL, Cyr61 protein: 100 ng/mL) Cyr61 protein (**a**, **e**: 100 ng/mL; I: 800 ng/mL); antiCyr61 antibody (ab) for 2 h (**d**: 100 ng/mL; **e**: 50 ng/mL, **i**: 50, 100, 200 ng/mL) before IL-6 and Cyr61 protein treatment. **h** Original magnification × 10. Values are means (± standard deviation) of at least three independent experiments. *p < 0.05, **p < 0.01, ***p < 0.001

Both IL-6 and Cyr61 protein enhanced FLS migration compared to the control group (Fig. 5a). Because Cyr61 is associated cell proliferation, we determined whether IL-6 and Cyr61 stimulate RA-FLS proliferation through analysis of cell proliferation. IL-6 and Cyr61 protein promoted FLS proliferation compared to the control group. (Fig. 5b). As shown in Fig. 4, IL-6 increased levels of the secreted Cyr61 protein and promoted Cyr61 protein synthesis. After confirming that IL-6- induced Cyr61 protein synthesis was blocked by the anti-Cyr61 antibody, decreasing the Cyr61 protein level (Fig. 5c), we observed an attenuation of IL-6-induced and Cyr61-induced increase in FLS migration by the anti-Cyr61 antibody (Fig. 5d, e), which was confirmed by the use of siRNA to knock down *CYR61* (Fig. 5f, g). The invasion data from the transwell assays also indicate that FLS invasiveness was increased by Cyr61 treatment compared to the control group (Fig. 5h) and was reduced by the neutralising antibody (Fig. 5i).

## Discussion

IL-6 plays a critical role in the development of RA [10, 15] and has been found to stimulate vascular endothelial growth factor production by vascular endothelial cells in affected joints, leading to joint swelling, synovial growth, and accumulation of synovial fluid [15]. Several studies showing that Cyr61 increases invasion and angiogenesis in several types of tumours led us to hypothesise that Cyr61 might be functionally linked to IL-6 [16–18]. Furthermore, pro-inflammatory cytokines, such as IL-17, have been recently shown to affect the expression of *CYR61* in FLSs [19]. Our results reveal that CYR61 mRNA expression and protein synthesis in FLSs from RA patients were enhanced by IL-6, and that both Cyr61 and IL-6 enhanced migration and invasion of RA-FLSs.

Joint destruction with synovitis is a characteristic of affected joints in RA. Pannus formation results in direct contact of FLSs with bone and cartilage tissues, leading to cartilage and bone destruction. The pannus invades cartilage, the surface of which is covered by FLSs, after which the local invasion of the cartilage matrix by the pannus starts [20]. We suspected that elevated levels of IL-6 in RA synovium might affect the FLS genotype and accelerate pannus formation. A transcriptome analysis was performed to identify genetic alterations of RA-FLSs after IL-6 treatment. In our study, we identified 315 DEGs between the RA samples and IL-6-treated RA samples, including 277 upregulated genes and 38 downregulated genes. A GO enrichment analysis revealed that the upregulated genes were mainly involved in blood vessel development, regulation of cell adhesion, and chemotaxis. These GO terms represented angiogenesis and regulation of epithelial cell migration that are associated with a cancer-like phenotype. In addition to TNF and the nuclear factor-kappa B (NF-κB) signalling pathway related to the inflammatory response, IL-6-induced FLS gene expression also regulates the MAPK signalling cascade, which is critical in the invasion and angiogenesis of cells. Our results suggest that increased IL-6 levels promote FLS phenotype transition to tumour-like patterns that are responsible for the vicious cycle of cytokine and chemokine production. These findings are consistent with the finding that IL-6 stimulates tumour-like proliferation of FLSs in RA [21] and indicate that tumour progression mechanisms could account for pannus formation and function.

The 38 downregulated DEGs found in our study are most closely associated with FOXO-mediated transcription. FOXO transcription factors regulate many cellular processes, including cell survival, apoptosis, and resistance to oxidative stress. In particular, FOXO proteins regulate bone cell survival, cell cycle, and proliferation and also participate in network control among different kinds of bone cells [22]. Given that our results show that IL-6 downregulated FOXO-mediated transcription in FLSs, further research on RA pathogenesis is warranted.

IL-6 acts through the JAK/STAT, MAPK, and PI-3 K/AKT pathways [23]. It binds to plasma membrane receptor complexes or to soluble receptor complexes to trigger its association with the signal-transducing gp130 [24]. Signal transduction involves activation of JAK kinases, leading to the activation of transcription factors of the STAT family, particularly STAT3 [25]. To determine which pathway is responsible for promoting the expression of *CYR61*, we used known inhibitors of several pathways. Although the canonical pathway of IL-6 is reported to be the JAK/STAT pathway, our results suggest that IL-6 altered Cyr61 protein synthesis mainly through the ERK 1/2 pathway. Although IL-6 and IL-6R are known to be important targets for RA, our results suggest that targeting IL-6 specific downstream signalling proteins could also be beneficial for RA therapy.

Based on our DEG results, we selected candidates such as c-Jun, NR4A1, ATF3, and EGR3 to determine whether they regulated IL-6-induced Cyr61 protein synthesis. Previous studies have suggested that the effect of IL-6 on target gene transcription may involve c-Jun [26]. Hence, we examined whether c-Jun was involved in the IL-6-induced increase in Cyr61 protein synthesis and found that knocking down *c-Jun* expression did not affect IL-6-induced Cyr61 protein synthesis. NR4A1 and ATF3 also did not show any significant effects.

Interestingly, we found that EGR3 modulated IL-6-induced Cyr61 protein synthesis through the ERK 1/2 pathway. EGR3 is a member of the early growth response (EGR) gene family of transcription factors that regulates a wide range of biological processes in response to growth factors, cytokines, and mechanical forces. In human foreskin fibroblasts lacking EGR3, transforming growth factor-beta 2 (TGF-β2) induction of the fibrotic genes [collagen alpha 1 (*COL1A1*), alpha-smooth muscle actin (*ACTA2*), *TGFB1*, connective tissue growth factor (*CTGF*), and plasminogen activator inhibitor-1 (*SERPINE1*) ] was significantly abrogated [27]. Furthermore, in human T cells, EGR4 and EGR3 interact with NF-κB to control the transcription of genes encoding inflammatory cytokines such as IL-2 and TNF-α, as well as intercellular adhesion molecule 1 [28]. Thus, EGR3 contributes to production of Cyr61 as a fibrotic or inflammatory mediator. Further studies are required to clarify the binding site for EGR3 on the *CYR61* gene.

As a secreted ECM protein, Cyr61 is considered to mediate cell proliferation, adhesion, and migration and act on pro-inflammatory molecules that induce the production of several cytokines and chemokines [29]. Moreover, activation of MMPs is essential for cells to migrate, through the rearrangement of ECM to facilitate cell migration [14]. Given its autocrine and paracrine features, increased secretion of Cyr61 by FLSs after IL-6 stimulation was found to promote proliferation and stimulate the expression of *MMP2*, suggesting a role of Cyr61 in the activation of FLS migration and invasion. These results support the hypothesis that expression of *CYR61* leads to hyperplasia and increased angiogenesis and invasion in joints in RA, leading to greater degrees of synovial inflammation and cartilage erosion. We confirmed that Cyr61 promoted IL-6 expression, thereby forming a positive feedback loop. Thus, specifically how IL-6 and Cyr61 create a positive feedback system requires further study. We also observed that IL-6 and Cyr61 led to an increase in FLS proliferation. However, in this study, we focused on migration and invasion of FLS. The mechanism of effect of IL-6 and Cyr61 on FLS proliferation requires further study.

**Fig. 6:**
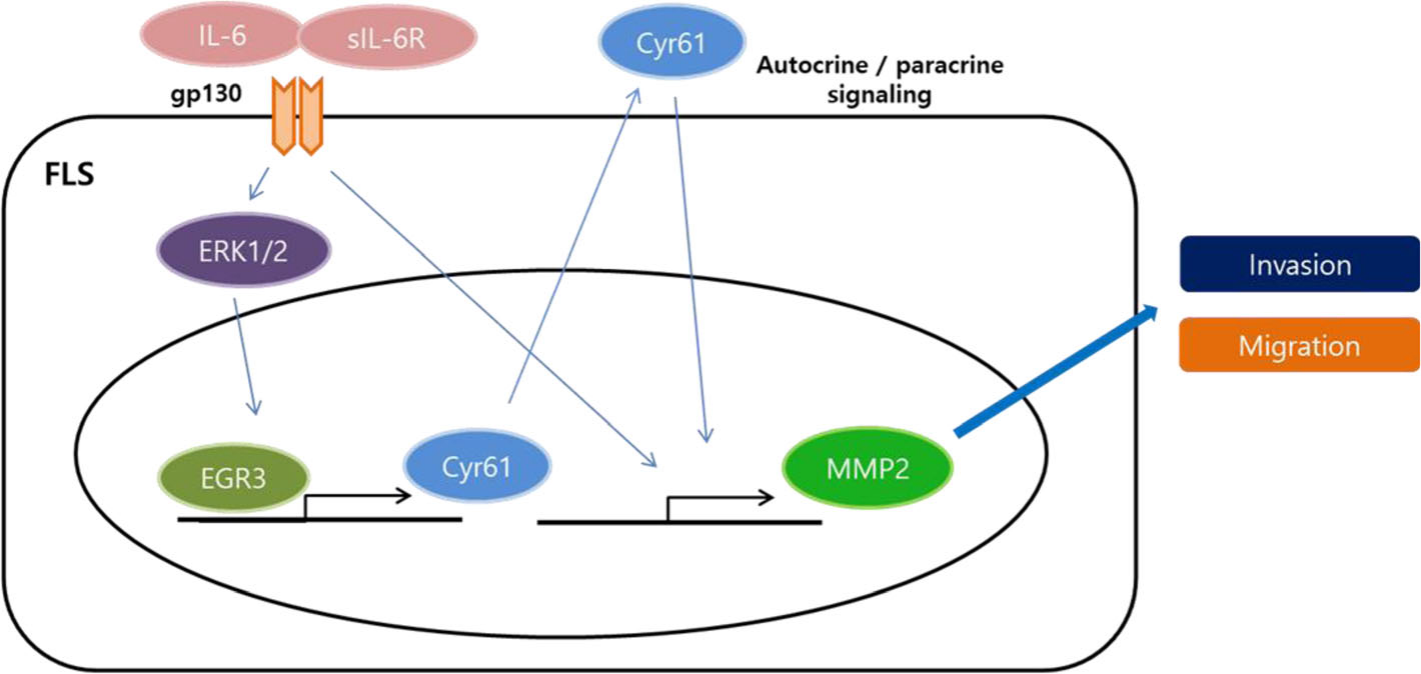
A schematic model for IL-6-stimulated *Cyr61* expression and its role in FLS migration and invasion. IL-6 and soluble IL-6 receptor complex stimulate *Cyr61* expression via the ERK/ EGR3 signalling pathway. Secreted Cyr61 protein activates FLSs in an autocrine or paracrine manner and the resulting increase in extracellular Cyr61 protein enhances FLS migration and invasion. IL-6, interleukin-6; FLS, fibroblast-like synoviocyte; ERK, extracellular signal-regulated kinase; EGR3, early growth response 3

To summarise, we report here that protein synthesis of Cyr61 was enhanced in the FLSs of RA patients compared to those from OA patients. Interestingly, IL-6 stimulated the expression of *CYR61* through the ERK/EGR3 pathway in RA-FLSs and modulated the expression of genes associated with angiogenesis, cell migration, and MAPK cascade. Cyr61 was produced and secreted into the ECM environment and acted on the FLSs in an autocrine/paracrine manner. *MMP2* expression was consequently enhanced and contributed to FLS migration and invasion. As a result, RA FLS migration and invasion were stimulated due to an enhancement of *CYR61* expression (Fig. 6).

## Conclusions

Our findings suggest that IL-6 regulated Cyr61 is a key player in FLS migration and invasion and eventually contributes to joint destruction in RA. Therefore, Cyr61 could be a potential therapeutic target for anti-IL-6 treatment of RA.

## Supplementary information

**Additional file 1: Fig. S1.** Transcription factors not involved in IL-6 induced Cyr61 protein synthesis. RA-FLSs transfected with either small interfering RNA (NR4A1 or ATF3) or siNC (control) (20 pmol/L) stimulated by IL-6 (20 ng/mL) for 2 h. Data are representative of at least three independent experiments. **Fig. S2.** Expression of IL-6 enhanced by Cyr61 secretion. The mRNA level of IL-6 stimulated by Cyr61 protein (100 ng/mL) for 2 h as determined via real time polymerase chain reaction. Values are means (± standard deviation) of at least three independent experiments. **p < 0.01.

## Data Availability

The datasets used and/or analysed during the current study are included in this article.
