# Correction to: Cyr61 synthesis is induced by interleukin-6 and promotes migration and invasion of fibroblast-like synoviocytes in rheumatoid arthritis

**DOI:** 10.1186/s13075-020-02381-y

**Published:** 2020-12-15

**Authors:** Changmin Choi, Wooseong Jeong, Byeongzu Ghang, Yonggeun Park, Changlim Hyun, Moonjae Cho, Jinseok Kim

**Affiliations:** 1 Department of Medicine, Jeju National University School of Medicine, Jeju, Republic of Korea; 2 Division of Rheumatology, Department of Internal Medicine, Jeju National University Hospital, Aran 13gil, Jeju, 690-797 Republic of Korea; 3 Department of Orthopaedic Surgery, Jeju National University Hospital, Jeju, Republic of Korea; 4 Department of Pathology, Jeju National University Hospital, Jeju, Republic of Korea; 5 Department of Biochemistry, Jeju National University School of Medicine, Aran 13gil, Jeju, 690-797 Republic of Korea

**Correction to: Arthritis Res Ther 22, 275 (2020)**

**https://doi.org/10.1186/s13075-020-02369-8**

Following publication of the original article [1], a typesetting error occurred in the spacing of text in Figs. 1a, d, f, Fig. 3f, Fig. 4a–c, e, f, Fig. 5a and f.

**Fig. 1:**
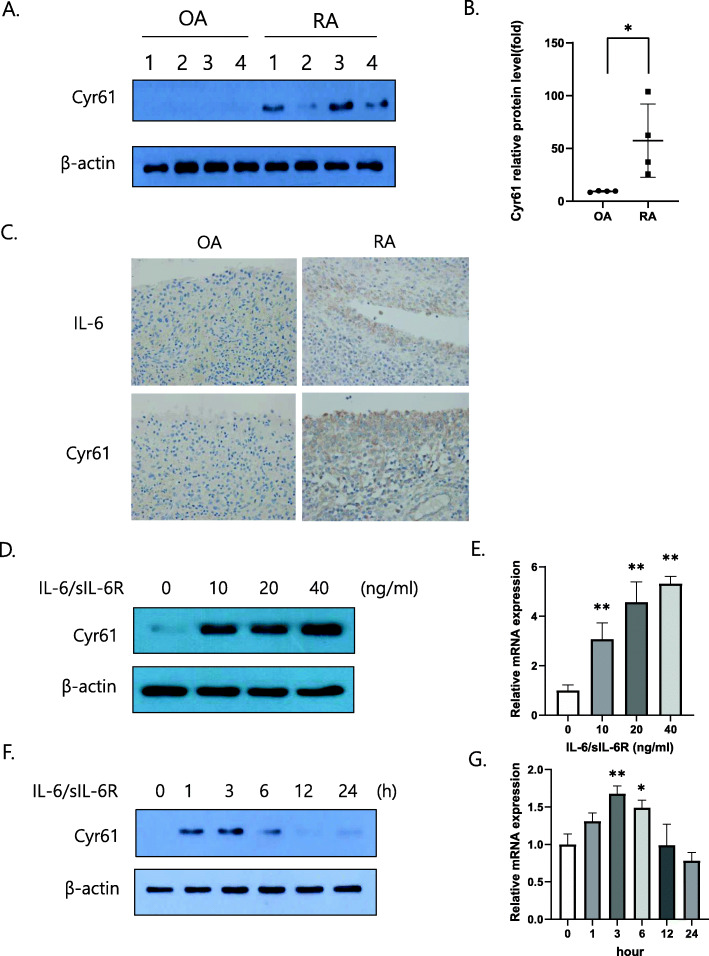
Expression of IL-6 and Cyr61 in fibroblast-like synoviocytes (FLSs). **a**, **b** FLSs from osteoarthritis (OA; *n* = 4) or rheumatoid arthritis (RA, *n* = 4) patients. *p < 0.05 vs OA. **c** Synovial tissues from OA or RA patients. Original magnification × 400. **d**, **e** RA FLSs stimulated by IL-6/sIL-6R for 2 h. **f**, **g** FLSs stimulated by IL-6/s IL-6R (20 ng/mL) for the indicated periods. **d**–**g** FLSs were incubated overnight in 1% FBS-containing medium before treatment with IL-6/sIL-6R. **a**, **b**, **d**, **f** Protein levels were determined by western blotting. **e**, **g** The mRNA levels of *Cyr61* were determined through real time polymerase chain reaction. Values are means (± standard deviation) of at least three independent experiments. *p < 0.05, **p < 0.01 vs untreated cells. IL-6, interleukin-6; sIL-6R, soluble IL-6 receptor; FBS, foetal bovine serum

**Fig. 3:**
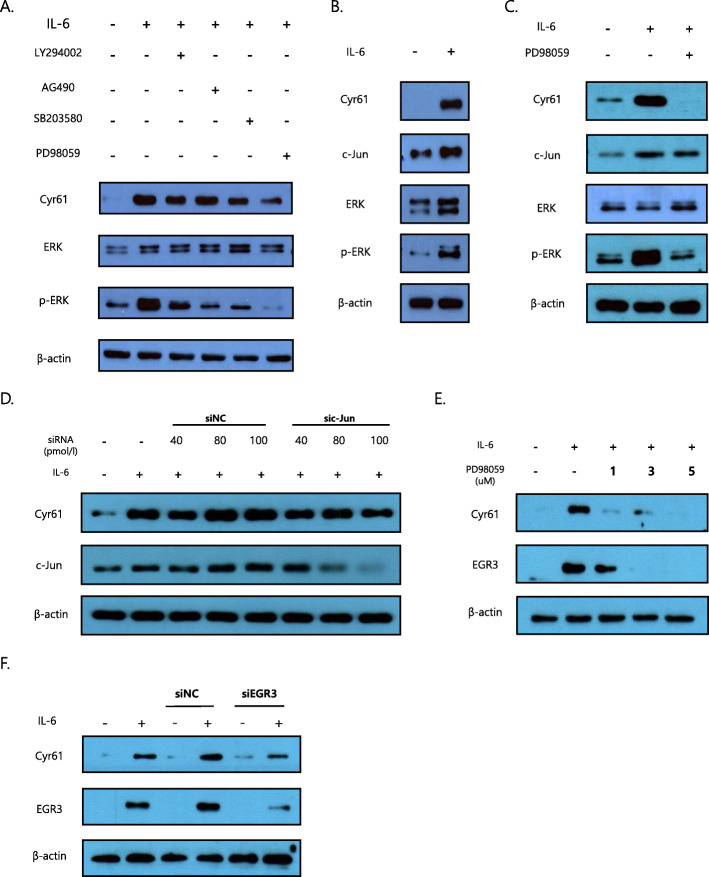
Signalling pathways involved in IL-6-regulated protein synthesis of Cyr61 in rheumatoid arthritis-fibroblast-like synoviocytes (RA-FLSs). **a**, **c**, **e** Cells were pretreated with inhibitors for 2 h before IL-6 (20 ng/mL) stimulation for 2 h. LY294002 (10 μM): PI3K/AKT inhibitor, AG490 (50 μM): JAK2/STAT3 inhibitor, SB203580 (10 μM): p38 MAPK inhibitor, PD98059 (1 μM): ERK inhibitor. **d**, **f** RA-FLSs transfected with either small interfering RNA (c-Jun or EGR3) or siNC (control) (20 pmol/L) stimulated by IL-6 (20 ng/mL) for 2 h. Data are representative of at least three independent experiments. **a–f** Protein levels were determined by western blotting. FLSs were incubated overnight in 1% FBS-containing medium before treatment with IL-6/sIL-6R

**Fig. 4:**
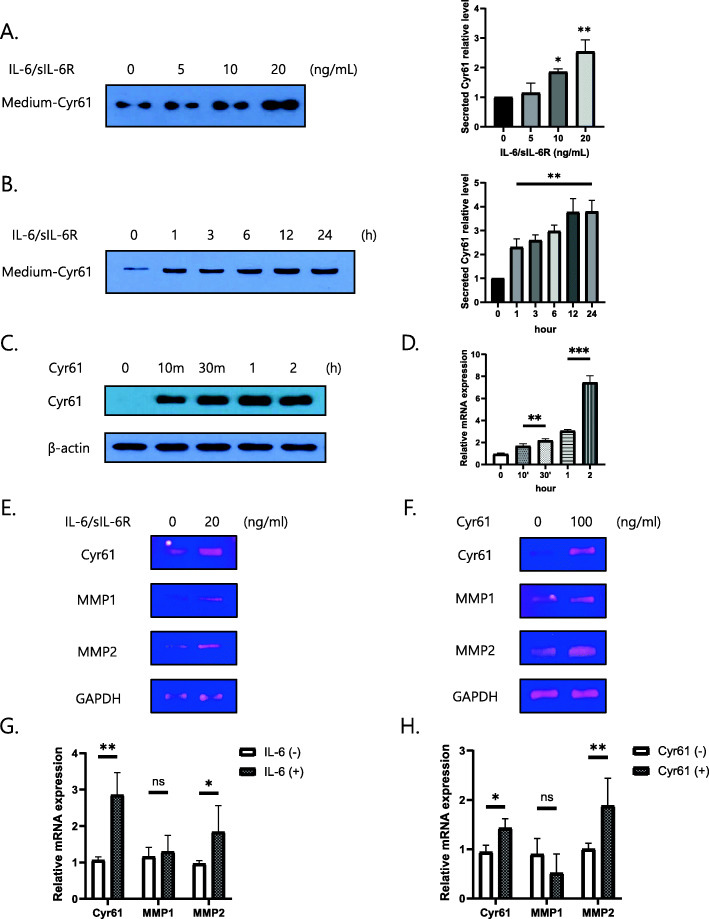
Cyr61 secretion induced by IL-6. **a**, **b** Extracellular protein levels of Cyr61 in culture supernatants of IL-6-treated RA-FLSs measured by western blotting. **b** IL-6 (20 ng/mL). **c**, **d** FLSs stimulated by extracellular Cyr61 (100 ng/mL) for indicated time periods. **c** Protein levels were determined by western blotting. **d** The mRNA levels of *Cyr61* were determined through real time polymerase chain reaction. **e**–**h** The mRNA levels of *Cyr61*, *MMP1*, *2*, and *GAPDH* induced by IL-6 (20 ng/mL) and extracellular Cyr61 protein (100 ng/mL) for 2 h. **e**, **f** The mRNA levels were determined by reverse-transcription polymerase chain reaction. **g**, **h** The mRNA levels were determined through real time polymerase chain reaction. Data are representative of at least three independent experiments. *p < 0.05, **p < 0.01, ***p < 0.001

**Fig. 5:**
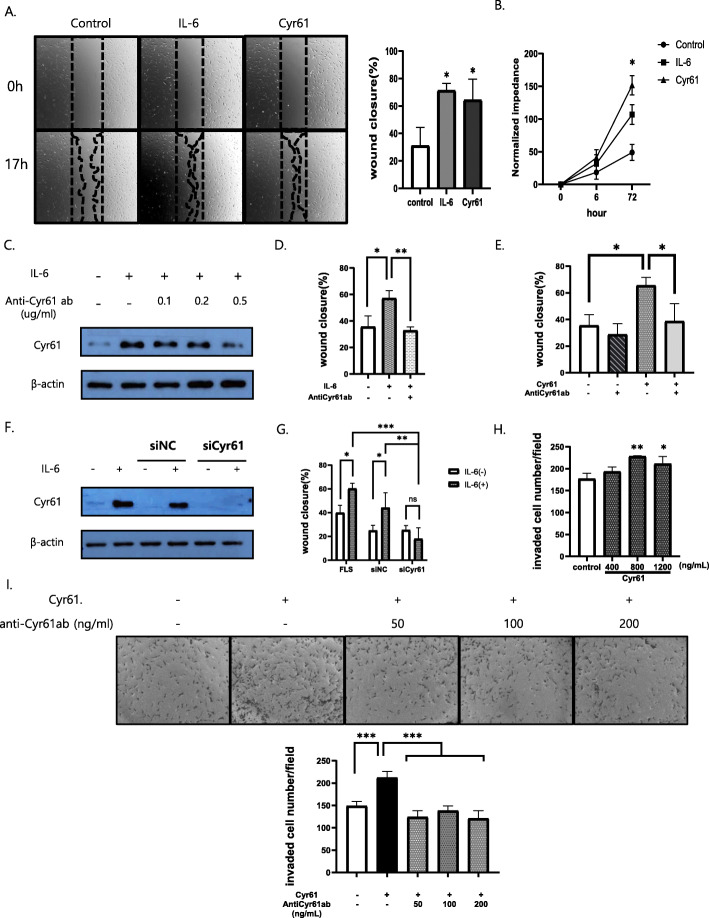
Migration and invasion of rheumatoid arthritis-fibroblast-like synoviocytes (RA-FLSs) promoted by IL-6 and Cyr61 secretion. **a**, **d**, **e**, **g** Wound-closure over 17 h. **b** ECIS proliferation analysis over 72 h. **c**, **f** Western blotting for Cyr61 protein detection. **h**, **i** Cyr61-stimulated invasion of RA-FLSs in transwells over 24 h ± antiCyr61 ab. **f**, **g** transfection with 20 pmol/L of small-interfering Cyr61 RNA (siCyr61) or siNC (negative control). IL-6 for 17 h (**a**, **d**, **g**: 200 ng/mL); IL-6 for 2 h (**c**, **f**: 20 ng/mL); IL-6 and Cyr61 protein for 72 h (**b**: IL-6: 200 ng/mL, Cyr61 protein: 100 ng/mL) Cyr61 protein (**a**, **e**: 100 ng/mL; I: 800 ng/mL); antiCyr61 antibody (ab) for 2 h (**d**: 100 ng/mL; **e**: 50 ng/mL, **i**: 50, 100, 200 ng/mL) before IL-6 and Cyr61 protein treatment. **h** Original magnification × 10. Values are means (± standard deviation) of at least three independent experiments. *p < 0.05, **p < 0.01, ***p < 0.001

The corrected figures are given in this correction article. The original article [1] has been corrected.
