# Supplementary figures and images for: Cyr61 synthesis is induced by interleukin-6 and promotes migration and invasion of fibroblast-like synoviocytes in rheumatoid arthritis
Source: Arthritis Res Ther. 2020 Nov 23;22:275. doi: 10.1186/s13075-020-02369-8 (PMC7685583; doi:10.1186/s13075-020-02369-8)

Supplementary figure 1.

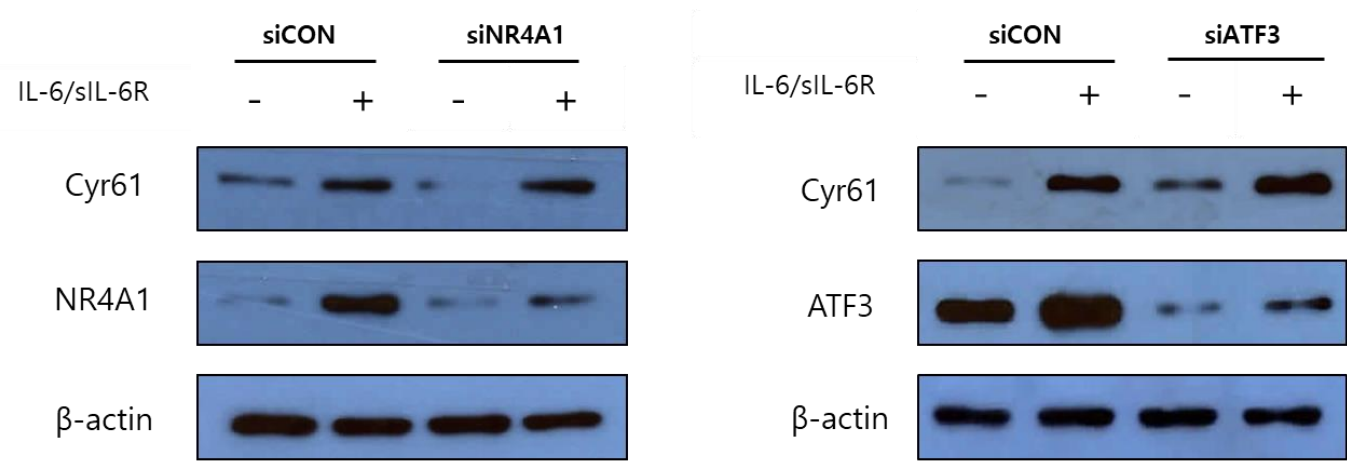

Supplementary figure 2.

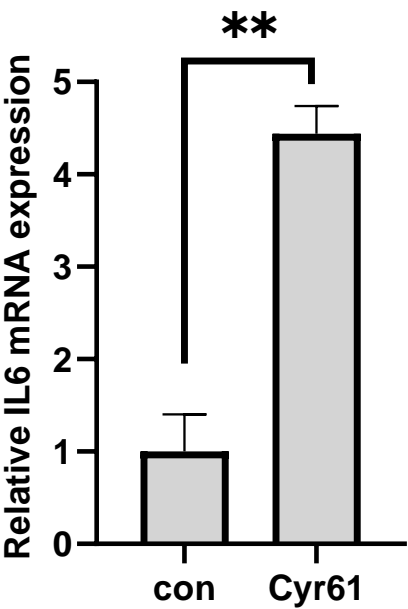

Supplement: Supplementary file 1 — Additional file 1: Fig. S1. Transcription factors not involved in IL-6 induced Cyr61 protein synthesis. RA-FLSs transfected with either small interfering RNA (NR4A1 or ATF3) or siNC (control) (20 pmol/L) stimulated by IL-6 (20 ng/mL) for 2 h. Data are representative of at least three independent experiments. Fig. S2. Expression of IL-6 enhanced by Cyr61 secretion. The mRNA level of IL-6 stimulated by Cyr61 protein (100 ng/mL) for 2 h as determined via real time polymerase chain reaction. Values are means (± standard deviation) of at least three independent experiments. **p < 0.01. [file 13075_2020_2369_MOESM1_ESM.pdf]
